# A retrospective cohort study comparing clinical outcomes and healthcare resource utilisation in patients undergoing surgery for osteomyelitis in England: a case for reorganising orthopaedic infection services

**DOI:** 10.5194/jbji-6-151-2021

**Published:** 2021-04-28

**Authors:** Jamie Ferguson, Myriam Alexander, Stuart Bruce, Matthew O'Connell, Sue Beecroft, Martin McNally

**Affiliations:** 1 Bone Infection Unit, Nuffield Orthopaedic Centre, Oxford University Hospitals NHS Foundation Trust, Oxford, OX3 7HE, UK; 2 Open Vie, Marlow, UK; 3 Health Economic and Outcomes Research Consultant, University of Otago, Dunedin, New Zealand; 4 Harvey Walsh, Cheshire, UK

## Abstract

**Aims**: An investigation of the impact of a multidisciplinary bone infection unit (BIU) undertaking osteomyelitis surgery with a single-stage protocol on clinical outcomes and healthcare utilisation compared to national outcomes in England. **Patients and Methods**: A tertiary referral multidisciplinary BIU was compared to the rest of England (ROE) and a subset of the 10 next busiest centres based on osteomyelitis treatment episode volume (Top Ten), using the Hospital Episodes Statistics database (HES). A total of 25 006 patients undergoing osteomyelitis surgery between April 2013 and March 2017 were included. Data on secondary healthcare resource utilisation and clinical indicators were extracted for 24 months before and after surgery. **Results**: Patients treated at the BIU had higher orthopaedic healthcare utilisation in the 2 years prior to their index procedure, with more admissions (p< 0.001) and a mean length of stay (LOS) over 4 times longer than other groups (10.99 d, compared to 2.79 d for Top Ten and 2.46 d for the ROE, p< 0.001). During the index inpatient period, the BIU had fewer mean theatre visits (1.25) compared to the TT (1.98, p< 0.001) and the ROE (1.64, p= 0.001). The index inpatient period was shorter in the BIU (11.84 d), 33.6 % less than the Top Ten (17.83 d, p< 0.001) and 29.9 % shorter than the ROE (16.88 d, p< 0.001). During follow-up, BIU patients underwent fewer osteomyelitis-related reoperations than Top Ten centres (p= 0.0139) and the ROE (p= 0.0137). Mortality was lower (4.71 %) compared to the Top Ten (20.06 %, p< 0.001) and the ROE (22.63 %, p< 0.001). The cumulative BIU total amputation rate was lower (6.47 %) compared to the Top Ten (15.96 %, p< 0.001) and the ROE (12.71 %, p< 0.001). Overall healthcare utilisation was lower in the BIU for all inpatient admissions, LOS, and Accident and Emergency (A&E) attendances. **Conclusion**: The benefits of managing osteomyelitis in a multi-disciplinary team (MDT) specialist setting included reduced hospital stays, lower reoperation rates for infection recurrence, improved survival, lower amputation rates, and lower overall healthcare utilisation. These results support the establishment of centrally funded multidisciplinary bone infection units that will improve patient outcomes and reduce healthcare utilisation.

## 1 Introduction

The incidence of osteomyelitis in western countries has risen over the past decades, which may be partially attributed to an evolution of the clinical diagnosis and partly driven by population ageing and increasing co-morbidities in the elderly (Kremers et al., 2015; Laurent et al., 2018; Ferguson et al., 2018). The burden of osteomyelitis on the healthcare system is significant. The surgery required to eradicate infection can be complex and patients with bone infection often suffer other chronic co-morbidities that require careful optimisation before surgery. Fractures complicated by early infection are associated with substantially higher financial costs as well as prolonged hospital stays. Infection was found to more than triple costs when complicating proximal femoral fractures (Pollard et al., 2006), and in the most severe open fractures, infection increased cost by 63 % and length of stay by 80 % (Olesen et al., 2017). A French study found osteomyelitis patients were hospitalised for an average of 17.5 d yr${}^{-1}$, with a 20 % risk of rehospitalisation (Laurent et al., 2018), with annual costs of treatment for bone and joint infections in France rising from EUR 259 million to 421 million between 2008 and 2013 (Grammatico-Guillon et al., 2012). Another study demonstrated that if complex orthoplastic surgery is required to manage osteomyelitis, with infection excision and free flap coverage, then the hospital is unable to recoup its costs, with an average loss of GBP 10 168 per patient (Shirley et al., 2018).

Early involvement of multidisciplinary teams (MDT) is recognised as being important in preventing complications and improving outcomes in those with osteomyelitis and fracture-related infection (FRI) (Grammatico-Guillon et al., 2012; Olesen et al., 2017; Ferguson et al., 2018; Vasoo et al., 2019; Metsemakers et al., 2020). Teams should include orthopaedic surgery, infectious diseases, plastic surgery, radiology, and in some instances also vascular surgery (Lew and Waldvogel, 2004). The new British Orthopaedic Association Standards for Trauma in FRI (British Orthopaedic Association, 2019) underline the importance of MDTs for managing infection. However, high costs have been reported as impeding treatment access for patients at risk of reinfection (Hackett et al., 2015). The current tariff system's inability to adequately remunerate hospitals undertaking this complex work further compounds this problem, with the majority of tariff payments failing to cover the actual costs incurred in managing complex cases (Shirley et al., 2018; Kendall et al., 2018). Consequently, many units remain unable to provide this service due to the costs of setting up and running such an MDT, despite national recommendations. The tariff remuneration disincentivises hospitals from developing regional bone infection units, with the most complex cases losing the most money. Currently, treatment varies across the nation, with much of the work undertaken emergently.

In many centres, surgery is staged and includes several procedures from debridement to reconstructive surgery (Rao et al., 2011), often performed in separate hospitals. Some countries, including France, have introduced successful models with centrally funded reference centres for bone and joint infections to address this complexity, but only following reforms brought in by central government (Laurent et al., 2018).

In this study we investigated a tertiary referral bone infection unit in England which manages chronic osteomyelitis and FRI. All cases are cared for by an MDT comprising orthopaedic and plastic surgeons, microbiologist/infectious disease physicians and radiologists with a special interest in bone infection. All cases are assessed jointly by all these specialties in an MDT clinic. Surgery is planned and performed in a single stage, using local antibiotic carriers to manage osseous dead space and commonly includes orthoplastic soft tissue reconstruction at the index procedure (Ferguson et al., 2017, 2019; McNally et al., 2017). In 2013, the BIU introduced a new antibiotic carrier called Cerament G (Bonesupport AB, Lund, Sweden) into the treatment protocol to facilitate this single-stage approach.

Currently little is known about the financial burden which osteomyelitis places on the NHS or the healthcare utilisation associated with its management. Whilst determining accurate cost data is difficult and outside the scope of this paper, this study aimed to determine whether a specialist centre can deliver improved clinical outcomes and reduced healthcare utilisation for patients treated surgically for osteomyelitis, as assessed using hard endpoint measures.

## 2 Patients and methods

This study observed patients for 2 years before and after surgery for osteomyelitis in all English hospitals. This allowed evaluation of healthcare resource utilisation and clinical outcomes and compared a specialist infection reference centre to the rest of England and a subset of other high-volume centres.

### 2.1 Database and reference site

This was a retrospective observational study based on patient-level data obtained from an existing secondary care database, the Hospital Episodes Statistics (HES) (further information is available online at http://www.hesonline.nhs.uk, last access: 1 April 2021). HES collects details of inpatient admissions, attendances to Accident and Emergency departments (A&E) and outpatient appointments at all NHS hospitals across England. It also includes cases managed in independent treatment centres and private hospitals, if funded by the NHS (NHS Digital, Hospital Episode Statistics, 2021). HES does not capture data on osteomyelitis treated in the private sector, meaning there is no source of accurate data for the number of osteomyelitis cases treated outside the NHS, though this will be a very small number each year. HES records all individual hospital admission spells, clinical information (e.g. diagnoses and procedures performed), patient characteristics (e.g. age and gender), and administrative data from the date of admission until the date of discharge.

As with any large-scale national database, the quality of the data is affected by many factors, such as the hospital coding process, data recording and searchability. Processes are in place to check the data quality, and although data are collected by the hospitals themselves, they are checked centrally, and the coding is independently audited regularly by unannounced visits to the hospital coding departments. Numerous published epidemiological studies in various clinical areas have evaluated clinical outcomes and healthcare resource utilisation using HES, which is considered a valid electronic health records database for use in research studies (Thorn et al., 2016a, b; Laudicella et al., 2016; Pennington et al., 2015: Sinha et al., 2013).

The data were downloaded from an NHS Digital portal in the form of a pipe-delimited text file. The two software packages to analyse the data were SQL Server Management Studio, version 15 (Microsoft, Redmond, Washington, USA), and Stata/SE, version 14.2 (Statacorp, College Station, Texas, USA). The data quality is checked via NHS Digital, who release data quality notes with each monthly release. The case series was selected by applying an M86 osteomyelitis diagnostic code during the observation time period. Next the osteomyelitis procedure codes were applied to arrive at the study cohort. This process was performed by the heath informatics company on our behalf (Harvey Walsh). The summary dataset was presented in Excel format.

In addition, mortality data are linked to HES via Civil Registry Data, which captures information on deaths occurring inside and outside of hospitals, including the underlying cause of death recorded on death certification.

### 2.2 Study population

The HES database was used to identify all patients with at least one diagnosis of osteomyelitis (identified using International Classification of Disease version 10 codes: M86*) recorded between 1 April 2013 and 31 March 2017. From this cohort, we identified all patients who underwent at least one surgical intervention to treat osteomyelitis during this time period (identified using surgical codes from the Office of Population Censuses and Surveys Classification of Surgical Operations and Procedures, 4th Revision). The first procedure for osteomyelitis identified in this time period was labelled as the *index procedure*. The time period between admission to hospital for the index procedure and subsequent discharge was labelled as the *index inpatient period*. Thus, the earliest surgical procedure during the observation period was labelled as the index procedure for the centre in which it was undertaken. All subsequent healthcare utilisation would therefore be linked to the index centre, irrespective of where it was received in England. All included hospitals had an orthopaedic surgery department and were staffed by qualified orthopaedic surgeons, though not necessarily those with a specialist interest in managing infection cases.

### 2.3 Inclusion and exclusion criteria

All cases with a primary diagnostic code of M86 for osteomyelitis AND who underwent surgery for this condition in the study period were included. Cases less than 18 years of age were excluded, as were all cases diagnosed with osteomyelitis that did not have surgery for this condition. Cases undergoing amputation for diabetic complications rather than osteomyelitis were also excluded.

### 2.4 Patient observation periods

To understand the burden of osteomyelitis on the NHS, healthcare utilisation data were also collected for the 24 months prior to index admission (the *pre-index period*). To evaluate healthcare utilisation after the index admission, data were also collected for 24 months following discharge after the index inpatient period (the *post-discharge period*). In contrast, the 24-month follow-up time period measuring clinical end points, such as mortality and amputation rate, was defined as starting from the day after the index procedure rather than from the discharge date.

The 2013–2017 inclusion period was chosen as being the most recent time period with adequate follow-up for infection outcomes (McNally et al., 2017). At the same time a new antibiotic-loaded bone graft substitute called Cerament G (Bonesupport AB, Lund, Sweden) was introduced in the UK, used to facilitate single-stage surgery for osteomyelitis, and which has been shown to be associated with a low infection recurrence rate in a previous series (McNally et al., 2016). The BIU has used local antibiotic carriers for managing osseous dead space in cavitary defects for many years as part of a published protocol (Ferguson et al., 2017; Mifsud et al., 2019). In cases without significant bone voids following infection excision (such as those with cortical osteomyelitis or those with segmental defects obliterated by acute bone shortening and gradual re-lengthening), local antibiotics may not have been used.

**Figure 1:**
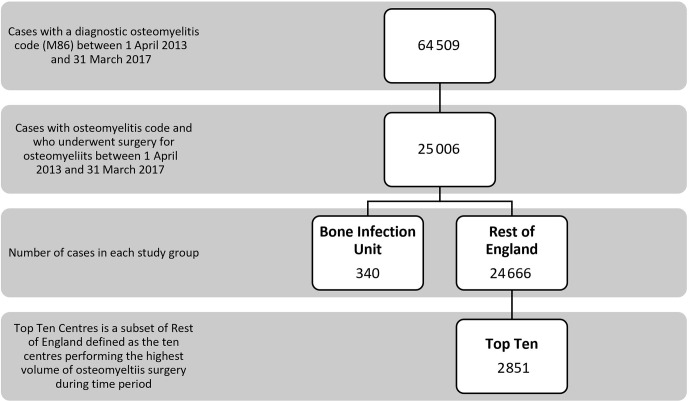
Flowchart of numbers included in the study and in each subgroup.

### 2.5 Subgroup analysis

Eligible patients were analysed as three subsamples, namely (1) *the bone infection unit* (BIU), all patients identified as receiving their first osteomyelitis surgery of the observation period at the Bone Infection Unit at the Nuffield Orthopaedic Centre, Oxford, UK, a specialist multidisciplinary centre specialising in managing osteomyelitis, (2) *the rest of England* (ROE), all other patients receiving their first osteomyelitis procedure of the observation period in England, excluding the BIU, and (3) *the Top Ten* (TT), a subset of the ROE patients as defined as the 10 centres undertaking the highest number of osteomyelitis surgeries during the study time period, excluding the BIU. The Top Ten were evaluated because it was considered that centres caring for more patients with osteomyelitis were most likely to be comparable to the BIU in terms of the diversity of the patient population and the care management process. It also allowed us to ensure that differences in healthcare utilisation were not simply due to volume of activity with efficiencies of scale. Whilst the BIU receives referrals from the local area, the majority of its referrals come from supra-regional and national referrals, which does mean many of its cases have more complex problems, often having previously had surgery that has not successfully controlled infection.

### 2.6 Demographics

Demographic data were extracted from the HES database, including gender, age at index surgery and time from diagnosis of osteomyelitis to first surgery. The number of osteomyelitis procedures was extracted during the index inpatient period for each day and for all days combined, in order to examine the impact of the single-stage approach (i.e. all surgeries performed in one operation) promoted at the BIU.

### 2.7 Outcome measures

Healthcare utilisation recorded for 2 years before and after the index admission included duration of inpatient hospital admissions to orthopaedic departments and all other healthcare inpatient departments, the number of outpatient attendances to orthopaedic and all other departments, and the number of attendances to A&E.

The clinical outcome measures included the mortality rate and the incidence of amputation in the 2 years following index surgery. For these clinical end points, the 24-month follow-up period started the day after the index procedure, to include all events occurring during the initial inpatient stay. Records of death were also extracted, including date and underlying cause of death.

### 2.8 Statistical analyses

Chi-squared tests with Yates' correction were used to compare categorical variables and a two-tailed t test used to compare quantitative variables, with mean and standard deviation described. P values < 0.05 were considered significant.

The rates of further osteomyelitis surgery and amputation in the post-discharge period were calculated by dividing the total number of these events by the number of patient years at risk during the follow-up period. Ninety-five percent confidence intervals (95 % CI) were estimated assuming an exact Poisson model. A Cox proportional hazard model was used with the BIU compared to the other groups for mortality and amputation rates, represented using Kaplan–Meier curves.

Statistical analyses were carried out using Stata v15 (StataCorp LLC), Microsoft SQL Server Management Studio v17.2 and Microsoft Excel 2010.

**Table 1:** Characteristics of patients included in the study and healthcare resource utilisation in the pre-index period (the 2 years before the index osteomyelitis procedure). Group One is the Bone Infection Unit (BIU). Group Two contains the 10 hospitals in England (excluding the BIU) with the highest number of osteomyelitis cases treated surgically in the study period. Group Three includes all remaining English hospitals treating osteomyelitis (excluding the BIU and the next 10 busiest hospitals). Figures represent the mean and (standard deviation) unless otherwise stated.

				P value	P value
	BIU	Top Ten	Rest of England	BIU vs.	BIU vs. Rest
Summary statistic	(n= 340)	(n= 2851)	(n= 24 666)	Top Ten	of England
*Patient characteristics*					
Mean age at index procedure, in years	50.83	56.60	56.09	< 0.0001	< 0.0001
	(16.70)	(21.75)	(23.88)		
Male (%)	69.12 %	67.84 %	64.22 %	0.676	0.0694
Time from first osteomyelitis diagnosis code to	49.12	65.14	53.83)	0.3231	0.7374
index procedure date, in days	(236.60)	(287.49)	(257.57		
*Resource utilisation in the pre-index period per patient*					
Mean number of admissions or attendances to	24.11	27.74	24.00	0.1358	0.9575
all hospital departments	(38.42)	(42.86)	(37.78)		
*Inpatient admissions*					
Mean number of admissions to orthopaedics	0.85	0.33	0.3	< 0.0001	< 0.0001
departments only	(1.48)	(0.95)	(0.84)		
Mean length of stay per patient for admissions	10.99	2.71	2.46	< 0.0001	< 0.0001
to orthopaedics department, in days	(37.59)	(11.17)	(12.35)		
Mean number of admissions to all hospital	2.32	6.13	4.62	0.0137	0.0375
departments	(3.44)	(28.46)	(20.38)		
Mean length of stay per patient for admissions	21.64	23.21	21.30	0.5512	0.8891
to all hospital departments, in days	(48.32)	(45.62)	(44.62)		
*Outpatient attendances*					
Mean number of outpatient attendances to	5.95	2.18	2.04	< 0.001	< 0.001
orthopaedics department	(7.31)	(6.14)	(5.15)		
Mean number of outpatient attendances to all	20.23	18.84	16.72	0.346	0.0098
hospital departments	(36.73)	(24.08)	(24.70)		
*Accident and Emergency attendances*					
Mean number of attendances to A&E	1.86	2.77	2.66	0.0112	0.0023
department	(2.57)	(6.55)	(4.83)		

## 3 Results

There were 64 509 patients with a diagnosis of osteomyelitis recorded between 1 April 2013 and 31 March 2017. Of that group, 25 006 patients (38.8 %) had at least one recorded operation to treat the osteomyelitis. Of the eligible patients undergoing osteomyelitis surgery during this time, 340 patients had the initial surgery at the BIU, and 24 666 patients had their initial osteomyelitis surgery in one of the other centres in the ROE. The subset of the Top Ten busiest hospitals from the ROE group included 2851 patients (see Fig. 1).

### 3.1 Patient population

Patients' mean age at index procedure was 50.8 years old (SD: 16.7) for the BIU, 56.6 (21.8) for the TT and 56.1 (23.9) for the ROE (p< 0.001 comparing BIU to TT and ROE). There was a preponderance of men in all cohorts (see Table 1).

**Table 2:** Activity during the index admission hospital stay (defined as the whole inpatient spell) for first-ever surgery for osteomyelitis during the observation period across the three groups. Figures represent mean and (standard deviation) unless otherwise stated.

				P value	P value	
	BIU	Top Ten	Rest of England	BIU vs.	BIU vs. Rest	
Summary statistic	(n= 340)	(n= 2851)	(n= 24 666)	Top Ten	of England	
Mean number of theatre visits	1.25 (0.60)	1.98 (2.71)	1.64 (2.20)	< 0.001	0.0011	
Mean number of procedures performed during inpatient index period	3.43 (1.92)	3.68 (3.22)	3.23 (2.94)	0.161	0.211	
Mean number of procedure codes performed during each trip to theatre during index admission	2.82 (1.76)	1.75 (1.18)	1.61 (1.14)	< 0.001	< 0.001	
Proportion of patients who underwent a plastic surgery procedure during inpatient index period	24.11 %	1.75 %	0.77 %	< 0.001	< 0.001	
Amputation rate during index admission	3.53 %	11.29 %	6.71 %	< 0.001	0.0261	
Mean length of inpatient stay per patient, in days	11.84 (12.12)	17.83 (27.25)	16.88 (27.43)	< 0.001	< 0.001	

**Figure 2:**
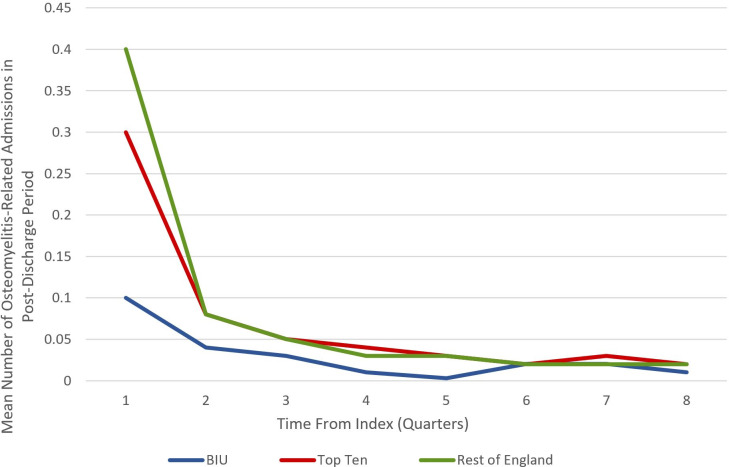
Mean number of osteomyelitis-related admissions during the 24-month post-discharge period at the BIU, Top Ten and the rest of England.

During the 2-year pre-index period there was no difference in hospital attendances or admissions across groups. However, in the BIU there were more admissions to orthopaedic departments, with a mean length of stay (LOS) 4.5 times longer than the ROE (10.99 d vs. 2.46 d, p< 0.001). There were also more orthopaedic outpatient attendances in the 2 years before the index procedure at the BIU (p< 0.001) although fewer A&E attendances compared to the ROE (p= 0.0023).

### 3.2 Index inpatient hospital stay for osteomyelitis surgery

During the index inpatient period, the BIU had lower mean theatre visits (1.25) compared to the TT (1.98, p< 0.001) and the ROE (1.64, p= 0.001; see Table 2). Although the total number of procedures undertaken was not different between the groups, it was clear that more procedures were undertaken during a single operative episode at the BIU (mean 2.82 procedures performed during single theatre) compared to 1.75 at the TT (p< 0.001) and 1.61 at the ROE (p< 0.001). The proportion of cases undergoing a plastic surgical procedure during the index inpatient period was higher in the BIU (24.11 %) compared to the TT (1.75 %, p< 0.001) and the ROE (0.77 %, p< 0.001).

The index inpatient period was 33.6 % shorter in the BIU (11.84 d) compared to the TT (17.83 d, p< 0.001) and a 29.9 % shorter stay compared to the ROE (16.88 d, p<0.001).

Furthermore, there was a lower incidence of amputation of the affected limb during the index inpatient stay at the BIU (3.53 %) compared to the TT (11.29 %, p< 0.001) and the ROE (6.71 %, p= 0.0261).

**Table 3:** Clinical outcomes during the 24-month post-discharge period following discharge from the index admission. Mortality and cumulative amputation rates are expressed from the day of the index procedure. Figures represent mean and (standard deviation) unless otherwise stated.

				P value	P value	
	BIU	Top Ten	Rest of England	BIU vs.	BIU vs. Rest	
Summary statistic	(n= 340)	(n= 2851)	(n= 24 666)	Top Ten	of England	
*Procedures*						
Mean number of osteomyelitis-related read-	0.24	0.56	0.66	0.0211	0.0140	
missions per patient	(0.58)	(2.55)	(3.15)			
Mean number of osteomyelitis procedures	0.23	0.57	0.63	0.0139	0.0137	
in post-discharge period per patient	(0.59)	(2.54)	(2.99)			
Mean number of osteomyelitis-related proce-	1.04	1.47	1.42	0.111	0.125	
dure codes performed per patient	(2.81)	(4.87)	(4.56)			
*Amputation*						
Amputation rate in post-discharge period only (percentage of original cohort)	2.94 %	4.67 %	6.00 %	0.189	0.0244	
Incidence rate of first amputation in post-	32.72	88.45	70.21	NR	NR	
discharge period per 1000 patient years (95 % CI)	(21.33–50.18)	(80.49–97.20)	(67.78–72.75)			
Cumulative total amputation rate during index admission and post-discharge period	6.47 %	15.96 %	12.71 %	< 0.001	< 0.001	
Incidence rate of amputation occurring at any	32.72	88.45	70.21	NR	NR	
time point per 1000 patient years (95 % CI)	(21.33–50.18)	(80.49–97.20)	(67.78–72.75)			
Rate ratio of amputation occurring at any time	Reference	2.70	2.15	< 0.001	0.001	
(95 % CI)		(1.75–4.41)	(1.40–3.47)			
*Mortality in the post-discharge period*						
Mortality rate (percentage of original cohort)	4.71 %	20.06 %	22.63 %	< 0.001	< 0.001	
Incidence rate of mortality per 1000 patient	24.29	112.73	128.72	NR	NR	
years (95 % CI)	(14.88–69.66)	(103.80–122.43)	(125.34–132.19)			
Mortality rate ratio (CI)	Reference	4.64	5.3	< 0.001	< 0.001	
		(2.83–8.17)	(3.26–9.27)			

### 3.3 Clinical outcomes in the post-discharge period

Patients treated at the BIU had fewer osteomyelitis procedures recorded during the 2-year post-discharge period (0.23) compared to the TT (0.57, p = 0.0139) and the ROE (0.63, p< 0.0137; see Table 3). This equates to a 34 % higher incidence of osteomyelitis surgery for the TT group and a 40 % higher incidence for the ROE compared to the BIU. In all the groups, the first 3 months following discharge contained the highest proportion of revision osteomyelitis procedures (accounting for 42.9 %, 52.6 % and 61.5 % of all the procedures performed in the 2-year follow-up period in the BIU, the Top Ten and the rest of England respectively) (see Fig. 2).

**Figure 3:**
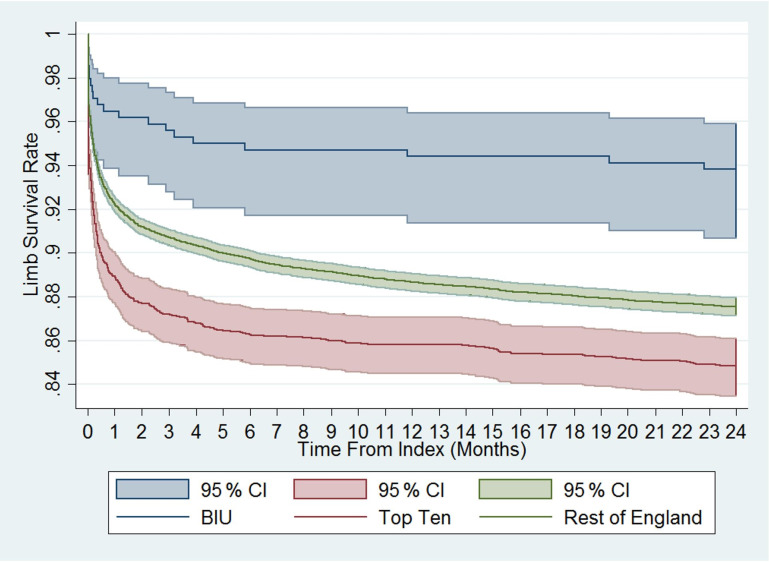
Cumulative limb survival rate. Time to first amputation from index procedure onwards. Comparison between the BIU, Top Ten and the rest of England.

The amputation rate as a percentage of the original group numbers during the post-discharge period was 2.94 % at the BIU, 4.67 % for the TT and 6.00 % for the ROE and was lower than the ROE (p= 0.024; see Table 3). The incidence rate became significantly different from 12 months after surgery and remained different at 24 months post-surgery (see Fig. 3). The cumulative total amputation rate, including amputations undertaken during the index inpatient period, was lower in the BIU (6.47 %) compared to the TT (15.96 %, p< 0.001) and the ROE (12.71 %, p< 0.001).

**Figure 4:**
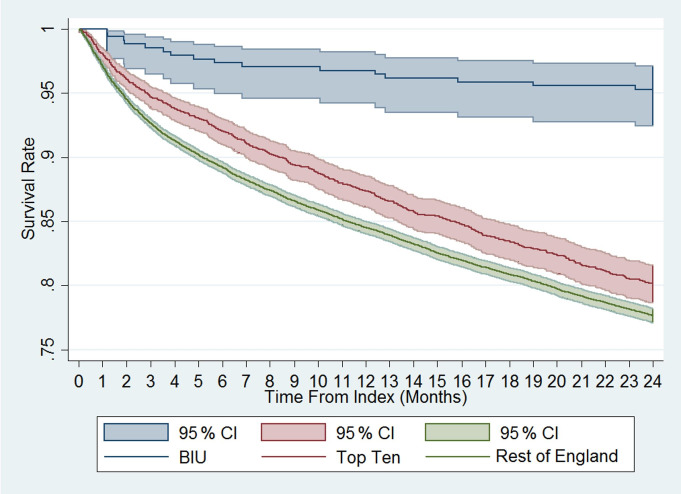
Overall survival following index osteomyelitis surgery; a comparison between the BIU, the Top Ten and the rest of England.

The mortality rate was more than 4 times lower in the BIU (16 deaths, 4.71 %) compared to the TT (572 deaths, 20.06 %, p< 0.001) and the ROE (4937 deaths, 22.63 %, p< 0.001; see Table 3 and Fig. 4). The mortality rate was different between all three groups from 3 months after surgery and remained so at 12 months and 24 months (Fig. 4). When death occurred within 24 months of surgery, osteomyelitis was the most commonly recorded condition occurring anywhere on the death certificate (including significant conditions contributing to the death) for deaths occurring in the TT (110 deaths) and the ROE (1132 deaths) but was the second most commonly recorded entry for the BIU (3 deaths).

**Table 4:** Healthcare resource utilisation per patient during the 24-month post-discharge period after index osteomyelitis surgery. Figures represent mean and (standard deviation).

				P value	P value	
	BIU	Top Ten	Rest of England	BIU vs.	BIU vs. Rest	
Summary statistic	(n= 340)	(n= 2851)	(n= 24 666)	Top Ten	of England	
*Inpatient admissions*						
Mean number of admissions to all hospital	2	5.82	4.74	0.0058	0.0072	
departments	(3.26)	(25.49)	(18.79)			
Mean length of stay per patient for admissions	12.92	23.46	23.8	< 0.0001	< 0.0001	
to all hospital departments, in days	(30.47)	(47.7)	(47.84)			
Mean number of admissions to orthopaedic	0.59	0.36	0.38	0.0027	0.0070	
departments	(0.97)	(1.37)	(1.43)			
Mean length of stay per patient for admissions	6.17	2.76	2.61	< 0.0001	< 0.0001	
to orthopaedic departments, in days	(17.4)	(12.38)	(12.15)			
*Outpatient attendances*						
Mean number of outpatient attendances to	5.22	2.80	2.94	< 0.0001	< 0.0001	
orthopaedic departments	(5.62)	(6.12)	(6.29)			
Mean number of outpatient attendances to all	19.77	26.15	21.17	0.0009	0.457	
hospital departments	(29.71)	(33.74)	(34.55)			
*A&E attendances*						
Mean number of attendances to A&E	1.11	2.12	2.15	0.0004	< 0.0001	
	(1.97)	(5.22)	(4.56)			

### 3.4 Healthcare resource utilisation

Overall patients treated at the BIU had lower healthcare utilisation during follow-up, with fewer inpatient admissions, shorter LOS and reduced A&E attendances (see Table 4). There were fewer outpatient attendances in the BIU compared to the ROE but not compared to the TT centres. However, when specifically looking at orthopaedic departments, the BIU had more admissions, longer LOS and more outpatient attendances compared to the other groups.

## 4 Discussion

We report the results of a large-scale, real-world study evaluating the burden and pathway of patients following surgery for osteomyelitis in England. Specifically, we compared outcomes and healthcare resource utilisation between other secondary care centres in England and a specialist bone infection unit that has adopted an MDT clinic and single-stage protocol.

This treatment pathway demonstrated an index LOS approaching one-third shorter than the TT and the ROE, with fewer theatre visits during admission. This does rely on more resource availability with higher proportions of cases requiring combined plastic surgical closure. Notably, there was a lower amputation rate in the BIU compared to the TT and the ROE, implying that more cases underwent limb salvage compared to the national average. This difference was more pronounced during follow-up, with the risk of undergoing amputation 1.9 times higher nationally and almost 2.5 times higher in the TT centres compared to the BIU. This carries a significant societal cost due to the high lifetime healthcare costs associated with rehabilitation and prosthetics for amputees. Amputation is thought to be at least 3 times more costly than limb salvage, and lifetime costs of amputation have been estimated to be above USD 500 000 (MacKenzie et al., 2007; Chung et al., 2009).

During follow-up the cases treated at the BIU had fewer reoperations for osteomyelitis, fewer admissions to hospital overall, shorter LOS and fewer A&E attendances compared to the TT and the ROE. Despite an overall lower healthcare utilisation in the post-discharge period at the BIU, there was a higher mean number of orthopaedic admissions and orthopaedic outpatient appointments. This finding may in part be explained by the higher mortality and amputation rates seen in the other groups. The follow-up in such cases would be significantly reduced as compared to cases undergoing successful limb salvage, which might make the mean number of orthopaedic attendances in the Top Ten and the rest of England look artificially low. This study revealed that patients required significant follow-up following osteomyelitis surgery. Those undergoing limb salvage surgery for osteomyelitis may need further ongoing intervention to treat other sequelae of infection, such as limb deformity, once the osteomyelitis has been addressed. The more complex cases require more extensive reconstruction and may require closer follow-up, for example if infection requires segmental excision. In this instance there may be a need to undertake reconstruction with an Ilizarov frame using bone transportation techniques. Such treatments are resource intense and require frequent additional outpatient activity to deliver this specialist care.

Most striking is the significant difference in mortality rates during follow-up. The risk of death was over 4 times higher outside the BIU. The difference persisted throughout the 2 years, with the survival curves continuing to diverge. Whilst it is not possible to fully explain the difference, it is interesting that osteomyelitis was the most common diagnosis found on the death certificate of those who died in the TT and ROE groups. The BIU population was statistically younger than the other cohorts (50.8 years vs. 56.1 years), but this could not account for the much higher mortality rate in isolation. A large Taiwanese study found that cases with osteomyelitis had a higher rate of mortality (increased incidence rate ratio of 2.29) compared to an age- and gender-matched cohort, with the effect remaining even 6 years after diagnosis (Huang et al., 2016). The higher rate of reoperation for osteomyelitis and subsequent amputation in the national cohort suggests that infection recurrence was more frequent in this group, which could have contributed to the higher mortality rates. What this does highlight is that nationally, surgically treated osteomyelitis has a worse 2-year survival than some forms of cancer, such as prostate, breast or Hodgkin's lymphoma (Office for National Statistics, 2018). Whilst the study demonstrated a higher death rate outside of the BIU during follow-up, it is not possible to directly attribute this difference to osteomyelitis on the information available.

The treatment protocol of the BIU relies on outpatient multidisciplinary clinical review to plan surgical management and combined orthoplastic surgery to allow immediate closure or flap coverage. Negative pressure wound therapy is not used. The use of absorbable antibiotic-loaded bone graft substitute for filling osseous dead space following excision is an important element of management as it has allowed the use of single-stage surgery in most cases. The use of absorbable antibiotic-loaded bone graft substitute (Cerament G, Bonesupport AB, Lund, Sweden) to manage osseous dead space in treating cavitary bone defects and FRI has shown encouragingly low infection recurrence rates (McNally et al., 2016; Ferguson et al., 2019). Furthermore, with the results of the recent OVIVA trial there has been a move away from long-term intravenous antibiotics, potentially reducing inpatient stays and improving cost effectiveness (Li et al., 2019; McMeekin et al., 2019).

This study has some strengths. Firstly, the patient population was well characterised, with all forms of surgically treated osteomyelitis included in the study (except for cases undergoing foot amputation for diabetic complications), including all cases treated for osteomyelitis in England. Secondly, HES data used for this study include all secondary centres in England and have been shown to be of high quality (NHS Digital, 2021; Thorn et al., 2016a, b; Pennington et al., 2015). Therefore, results may inform public health decisions at a national level. The 2-year follow-up is relatively long for assessing healthcare utilisation, although in the case of osteomyelitis this follow-up timeframe may not capture all recurrent disease because late recurrence is well recognised. However, it is accepted that a minimum of 2-year follow-up should identify over 90 % of all recurrences (McNally et al., 2017).

There are also some limitations that need consideration. Data on the severity of osteomyelitis were not available, which might impact clinical outcomes and healthcare resource use. We note that the BIU population was statistically younger, and one possible explanation for this difference might be that older, frailer patients might be less inclined to travel to the BIU for consultation as compared to the younger cohort referred from other national hospitals. Nevertheless, the pre-index LOS in orthopaedic departments in the BIU cohort was higher than the other groups, which might suggest these cases were more complex to begin with. Secondly, the study design meant that the first procedure for osteomyelitis in the observation period was used as the index procedure for each centre. If this surgery fails, successful revision surgery at a second centre will not be attributed to the second centre. Multiple previous unsuccessful treatment tends to make final definitive surgery more difficult and encourages the development of multi-resistant organisms with fewer antimicrobial options. Many of the cases that are seen in the BIU have had multiple previous unsuccessful procedures to cure infection in other centres before being referred in. Therefore, these cases are not being captured as having been treated in the BIU in this analysis.

It is not possible to single out one isolated element of the protocol as being the sole reason to explain the difference in outcomes between the groups. Successful management of osteomyelitis requires delivery of several interdependent elements of treatment. It is clear that the use of multidisciplinary clinics, pre-operative planning based on appropriate cross-sectional imaging, the availability of regular orthoplastic operating lists, the avoidance of negative pressure vacuum dressings (Yusef et al., 2013; Birke-Sorensen et al., 2011) and the management of osseous dead space using bioabsorbable local antimicrobial delivery all facilitate the delivery of single-stage surgery with all the resultant benefits described. This combination provides a “care bundle” which has proved successful in this condition. Further studies are required to identify the most important elements of care that result in reduced health care utilisation and improved patient outcomes.

Our data strengthen the argument for the establishment of appropriately funded specialist infection reference centres to allow delivery of this model of care. This model has successfully been adopted in France, leading to better patient care (Ferry et al., 2019). Review of the current tariff system is urgently needed to ensure that centres become financially viable. The higher costs associated with running specialist MDT centres to treat bone infection may be justified given the improved patient outcomes and reduced healthcare utilisation following surgical intervention demonstrated in this paper. This is particularly pressing considering the recommendation of the Getting it Right First Time Report (GIRFT, 2015) to centralise complex work to improve outcomes.

In conclusion, the management of osteomyelitis is demanding and costly. However, when this condition is managed in an MDT specialist setting as described, the resultant benefits included reduced hospital stays, lower reoperation rates for infection recurrence, improved survival, lower amputation rates, and lower overall healthcare utilisation during follow-up. These results support the establishment of a centrally funded, multidisciplinary bone infection unit that will improve patient outcomes and reduce healthcare utilisation.

## Data Availability

Information on how and where to access underlying research data can be found in Sect. 2.
